# The utility of the implementation science framework “Integrated Promoting Action on Research Implementation in Health Services” (i-PARIHS) and the facilitator role for introducing patient-reported outcome measures (PROMs) in a medical oncology outpatient department

**DOI:** 10.1007/s11136-020-02669-1

**Published:** 2020-10-21

**Authors:** Natasha A. Roberts, Monika Janda, Angela M. Stover, Kimberly E. Alexander, David Wyld, Alison Mudge, the ISOQOL PROMs/PREMs in Clinical Practice Implementation Science Work Group

**Affiliations:** 1 Queensland University of Technology, Kelvin Grove, QLD Australia; 2 Royal Brisbane and Women’s Hospital, Herston, QLD Australia; 3 Centre for Health Services Research, The University of Queensland, Woolloongabba, QLD Australia; 4 Department of Health Policy and Management, University of North Carolina at Chapel Hill, Chapel Hill, NC USA; 5 Lineberger Comprehensive Cancer Center, University of North Carolina at Chapel Hill, Chapel Hill, NC USA; 6 School of Clinical Medicine, University of Queensland, St Lucia, QLD Australia

**Keywords:** Patient-reported outcome measures, Quality of life, Implementation science, Clinical practice, Routine care

## Abstract

**Purpose:**

We evaluated the utility of the implementation science framework “Integrated Promoting Action on Research Implementation in Health Services” (i-PARIHS) for introducing patient-reported outcome measures (PROMs) into a medical oncology outpatient department. The i-PARIHS framework identifies four core constructs for implementation, including Facilitation, Innovation, Context and Recipients.

**Methods:**

A pilot study used the i-PARIHS framework to identify PROM implementation barriers and enablers to inform facilitation support strategies, such as training clinicians and staff, workflow support, technical support and audit and feedback. Pre- and post-implementation surveys were completed by 83 and 72 staff, respectively, (nurses, doctors and allied health), to assess perceived knowledge, enablers, barriers and utility of PROMs; and acceptability of the PROM intervention was also assessed post-implementation.

**Results:**

Important barriers included time constraints and previous experiences with technology. Enablers included good leadership support and a culture of learning. Facilitation strategies were used to overcome barriers identified in the i-PARIHS core domains. Compared to before the intervention, staff surveys showed improvement in perceived usefulness, perceived understanding and interpretation skills for PROMs. Staff perceptions about lack of time to use PROMs during visits remained a major perceived barrier post-implementation.

**Conclusion:**

The i-PARIHS framework was useful for guiding the implementation of PROMs in routine oncology care. The four core i-PARIHS constructs (Facilitation, Innovation, Context and Recipients) identified factors that directly impacted implementation, with Facilitation having a particularly important role to overcome these barriers. Oncology clinics and health systems considering implementing PROMs should consider having a dedicated Facilitator available during PROM implementation.

## Background

In clinical trials, patient-reported outcome measures (PROMs) improve communication between patients and clinicians, resulting in quality of life and survival benefits [1–3]. Implementation strategies are key to translating health innovations into practice [4], but little is known about the optimal implementation strategies for PROM integration in medical oncology outpatient departments.

Care delivery transformation within the clinical environment of an outpatient oncology clinic is complex [5–7]. This complexity comes from the many levels of the larger health ecosystem [8] into which outpatient oncology wards are embedded [9], as well as the diverse needs of cancer patients undergoing a multitude of treatment protocols and follow-up [10]. Teams of multidisciplinary health professionals may be involved in each patient’s care and they may have competing priorities with diverse knowledge, attitudes and beliefs [11, 12]. In randomised controlled trials (RCTs) testing the value of PROMs, the clinical trial coordinator often played an invisible role facilitating uptake of the PROM innovation with patients and staff teams (e.g. conducting training sessions and running clinic-level reports) [13, 14]. However, in routine clinical practice, there may not be investment in a dedicated implementation support role to support this complex process [15]. To implement a new intervention into this type of an environment requires insight into the interplay of the innovation (PROMs), the context and setting and the people and roles involved [16].

The multiple stakeholders required to implement PROMs include patients, families, nursing staff, administrative staff, medical staff, allied health professionals and records managers, all need to be engaged in using PROMs [5]. Their involvement is required to ensure initiating, handling, interpreting, and responding to PROMs as part of routine care delivery [17, 18], thus making this a complex healthcare intervention [19].

We used the implementation science framework “Integrated Promoting Action on Research Implementation in Health Services” (i-PARIHS) to examine factors impacting the use of the PROM version of the Common Terminology Criteria for Adverse Events (PRO-CTCAE) [20] during patient care visits.

i-PARIHS is a framework specifically designed for implementing complex interventions in healthcare [21] and is unique in emphasising the role of a Facilitator as the central construct of successful implementation. Like other determinant frameworks in implementation science [22], i-PARIHS can be used to help understand and overcome the multiple influences impacting implementation. Based strongly on experiential learning and empirical evidence, whilst drawing on a range of behaviour change theories, i-PARIHS can be used to plan, guide and understand complex implementation in healthcare [23]. This paper reports on a pilot implementation case study using the constructs of i-PARIHS (see Box 1).

The aims of this paper are to

1. describe this case study of PROMs implementation in routine oncology practice using the i-PARIHS framework [21], to better understand what conditions are critical to enable integration of this intervention into the clinic;
2. describe the Facilitator role and how it was used to overcome barriers to PROM implementation;
3. determine changes in clinician and staff perceptions of acceptability [4] and knowledge of PROMs, and perceived skills in interpreting and using PROMs with patients and
4. summarise lessons learned to inform implementation, sustainability, scale and spread of future PROM projects.

Box 1: The intervention design The PROMs intervention consisted of patients completing the PRO-CTCAE in the waiting room of two oncology clinics in Australia, and clinicians reviewing patient responses and acting on burdensome symptoms. The core-set PRO-CTCAE measures cancer-specific symptoms such as constipation, diarrhoea, nausea, vomiting, shortness of breath, fatigue, anxiety, low mood, pain, peripheral neuropathy, and other symptoms [9]. Patients completed the PRO-CTCAE items via a touchscreen workstation in the waiting room. A paper copy of the patient’s PROM report was automatically printed, then immediately available to the care team for use during the visit. The PROM report was also imported into the patient’s scanned electronic medical record for future reference. The immediate availability of standardised symptom reports has been shown in RCTs to improve symptom detection, more consistent management of symptoms, quality of life and mortality [13].

Implementation took place in two oncology outpatient clinics in a large metropolitan hospital in Queensland, Australia. This hospital provides oncology services for the metropolitan area as well as specialist referral services for patients across the state.

## Methods

### Implementation framework

To comprehensively understand PROM implementation issues presented in the literature [25–29], and to inform selection of an implementation framework, we previously undertook a scoping review of systematic reviews [19]. This review identified key themes of (1) engaging clinicians, (2) integration of technology for PROMs and (3) strategies to respond to PROMs data [14]. It was identified that these themes correspond to core constructs of the i-PARIHS framework (*Recipients, Innovation* and *Context*), and so it was chosen as the guiding framework for our pilot study [30]. In this report, we present i-PARIHS terms in *italics*.

Each core construct of i-PARIHS has a sub-set of potential factors that can become enablers or barriers for implementation that can be addressed using *Active Facilitation,* which has been demonstrated to increase uptake of research evidence in primary care [24, 31, 32] and is “how” the implementation happens. Central to the i-PARIHS framework, *Facilitation* is “the construct that activates implementation through assessing and responding to characteristics of the *Innovation* and the *Recipients* (both as individuals and in teams) within their *Context*ual setting” [21, p. 6].

For this study, an internal *novice Facilitator* (NR), an oncology nurse experienced as a clinical trial coordinator, conducted study activities pre-, during- and post-implementation. Consistent with the concept of the *Facilitator’s journey* [30], the *novice Facilitator* engaged with a *expert Facilitator* (AM) and a local multidisciplinary peer group of clinician–implementers for reflection (and peer learning) every 1–2 months during the implementation. She also had structured academic support from a research team with university academic affiliations to the setting.

The scoping review of systematic reviews [14] theme of engaging with clinicians aligned with the i-PARIHS *Recipients* construct of “who” receives the intervention. Whilst the *Recipients* included clinicians, patients and health administration staff, this case study focuses on the views of the clinical staff, as the group who reviewed the PROM, interpreted patients’ responses and responded to achieve the anticipated clinical outcomes of the intervention. Clinical staff in the setting included nurses, doctors (specialist oncologists and trainees) and a range of allied health professionals, including dietitians, physiotherapists, occupational therapists, speech therapists, psychologists and social workers.

Within the i-PARIHS framework, potential *Recipient* enablers and barriers include factors, such as *motivation*, *value & beliefs*, *clinical consensus*, *local opinion leaders*, *existing data sources*, *skills and knowledge*, *time and resources*, *learning environment*, *collaboration and teamwork*, *power and authority* and *professional boundaries and networks* [21].

In the scoping review of systematic reviews [14], the theme of technology integration for PROMs aligned with i-PARIHS’s construct of the *Innovation.* This is “what” is implemented and is described in more detail in Box 1. Potential barriers and enablers within the *Innovation* construct include *underlying knowledge sources*, *clarity*, *degree of fit (compatibility or contestability)*, *degree of novelty*, *likely boundaries*, *trialability* and *relative advantage* [21].

The scoping review of systematic reviews theme [14] of strategies to respond to PROMs data aligned with i-PARIHS’s *Context,* “where” the implementation takes place and encompasses the characteristics of the setting.

Enablers and barriers considered under the *Context* domain included characteristics of the inner and outer context. *Inner Context* factors included *formal and informal leadership support*, *culture*, *past experience of change*, *mechanisms for embedding change and learning* and *evaluation and feedback processes. Outer Context* factors included *organisational priorities*, *structure*, *leadership and senior management support*, *systems and processes*, *culture*, *history of innovation and change,* and *absorptive capacity* (the ability to absorb change in the *Context*) [21].

### Study design

This study used a descriptive mixed methods case study design nested within a prospective trial of PROMs implementation in two oncology clinics [33, 34]. The research protocol of the trial is presented elsewhere [35].

### Data collection and analysis

Qualitative data were coded by the *Facilitator* (NR) [35] using a content analysis [36], informed by the i-PARIHS framework [21]. Data collected by the *Facilitator* over a 12-month period was informed by the research protocol. These data included field notes comprising structured observations of activity for context assessments and process mapping [21], weekly case report forms and transcribed quotes from *Recipients* as memos. Results from the pre-implementation findings had been previously shared (and amended for accuracy) with *Recipients* to plan the implementation and during the iterative evaluation cycles, then also reviewed by staff post-implementation. Additionally, free text responses on questions about perceived knowledge, perceived usefulness and perceived enablers and barriers in a clinician survey completed pre- and post-implementation were included as a qualitative dataset [35]. These qualitative data were collated for the evaluation by NR, and informed discussions with an *expert Facilitator* (AM).

To determine if each factor was a barrier or enabler from the *Facilitator* perspective, the *novice Facilitator* (NR) and *expert Facilitator* (AM) separately identified and rated factors along a visual continuum of barrier to enabler, and then combined their ratings by consensus. Consensus discussions were summarised into templated tables, and illustrative examples of facilitation strategies were agreed on by discussion. Final tables were reviewed for face validity by a third rater, the lead senior academic team member not directly involved in the health service (MJ). Whilst we recognise that such judgements are inherently subjective, potential biases on the part of the *Facilitator* (NR) were thus mitigated through a number of mechanisms, including the cycles of evaluation with participants, reflexive monitoring, expert facilitation discussions and collaboration with a wider academic team.

Quantitative data included a survey adapted from Rouette et al. [35, 37] used to elicit clinician and staff perceptions of PROM acceptability [34], knowledge and utility of PROMs, and enablers and barriers. Surveys were distributed to nursing, medical and allied health professionals involved in providing care to patients, before and after implementing the intervention. Data were summarised by discipline group, reporting the proportion of respondents who agreed or strongly agreed with each statement and are presented in a table for comparison.

The potential conflict of interest that could come from the *novice Facilitator’s* relationship with participants was addressed in the design of the study, including the ethical considerations that were integrated into the study protocols. For the anonymous staff surveys, participation was considered consent and staff could decline participation in surveys simply by not completing them. During the implementation phase, staff in-service education was provided, marketing flyers distributed and information sheets provided. These resources explicitly stated that participation was voluntary and staff could opt out at any time in writing on case report forms, or by simply not participating in the implementation work.

## Results

### Barrier and enabler ratings from the *Facilitator* perspective

Tables 1, 2, 3 show the consensus ratings of the novice and expert *Facilitators*, addressed for each i-PARIHS construct. Strategies used to overcome barriers are discussed in the text.

**Table 1:** Consensus expert and novice facilitator ratings, with examples, of i-PARIHS constructs in the *Recipients* domain as a barrier or enabler

i-PARIHS recipient constructs	Rating					Example
	Barrier			Enabler		
Motivation			X			Variation in perceived utility of PROMs within and between disciplines
Values/beliefs				X		Strong professional values of person-centred care amongst staff groups
Local opinion leaders				X		Over the course of the project, peer champions for PROMs emerged
Existing data sources		X				Lack of local data/reporting processes for patient symptoms
Skills and knowledge			X			Variable knowledge of PROMs within and between disciplines
Time and resources	X					Staff feeling pressured with competing tasks and priorities
Collaboration and teamwork		X				Professional silos and discipline-specific work practices
Power and authority				X		Project support by Medical Director as co-investigator
Professional boundaries and networks		X				Limited communication between disciplines, expressed concerns that other disciplines may not engage leading to disproportionate burden on some staff

**Table 2:** Consensus expert and novice facilitator ratings, with examples, of i-PARIHS constructs in the *Innovation* construct were a barrier or enabler

i-PARIHS *Innovation* constructs	Rating					Example
	Barrier			Enabler		
Clarity				X		PRO-CTCAE is a simple clinically relevant tool
Compatibility/fit		X				PROM completion and filing did not initially fit easily into existing workflows
Novelty			X			Staff varied in embracing new technology and concepts
Trialability				X		Commencing in single clinic permitted troubleshooting and reduced disruption and burden
Relative advantage					X	Credible recent research findings supported use. Support by the *Facilitator* assisted staff who were not confident in relying on the PROM information
Boundaries	X					Interfaces between electronic and paper records, patient-held and health system-held records

**Table 3:** Consensus expert and novice facilitator ratings, with examples, of whether i-PARIHS constructs in the *Context* as a barrier or enabler

i-PARIHS *Context* constructs	Rating					Example
	Enabler			Enabler		
Formal and informal leadership networks				X		Project lead had credibility and moderate support from medical and nursing leadership and peers
Culture			X			Competing priorities of throughput and patient-centred care leading to staff tension and competing priorities
Past experience with change		X				Staff had experience with previous unsuccessful initiatives that became burdensome
Mechanisms for embedding change		X				Previous changes had often failed to be sustained
Evaluation and feedback processes				X		Staff had some familiarity with audit and feedback, e.g. safety and quality audits
Learning environment				X		Available in-service education opportunities for staff; strong emphasis on research evidence
Organisational priorities			X			Efficiency and risk were highlighted as important
Leadership and senior management support			X			Research funding provided some credibility and funding discretion, but not seen as core activity
Structure and systems			X			Large oncology service; disciplines have separate reporting; complex interface with existing record systems
Absorptive capacity				X		Staff were accustomed to accommodating practice changes (e.g. frequent updates to medical protocols) often through “workarounds”
Learning networks		X				Limited connections with other services that were successfully using PROMs

Features of the *Recipients* (focussing on clinical staff) are summarised in Table 1. The *Facilitator* used educational strategies (e.g. training sessions with clinicians and staff) to introduce PROMs to clinicians and staff and how to interpret patient responses. An identified barrier was a concern that colleagues would not engage with PROMs, which could lead to disproportionate burden on some staff or even failure of the intervention. The *Facilitator* used open dialogue to alleviate these concerns.

Table 2 describes the *Innovation* domain. The *trialability* (the ability to test the *Innovation* at a small scale) improved the *degree of fit* (how well the *Innovation* integrated into workflows), by being flexible and using iterative implementation, with the *Facilitator* supporting each step of the process (e.g. patient report completion, report generation, filing and interpretation) through hands-on technical support and contingent problem solving. Through consultation with all disciplines as well as “hands-on” contingent assistance, the *Facilitator* was able to model and teach these changes as the implementation progressed. The PROMs intervention was reported by staff on surveys as having *relative advantage*.

Table 3 summarises features of the local and organisational *Context* that influenced implementation. Staff said they were concerned that the health service would not have the resources to respond to additional patient needs identified through the PROM information. The *Facilitator* needed to maintain momentum by intermittently providing PROMs reports at clinic level, and ensuring they were consistently transferred into the EMR record, until the workflows were established to ensure staff confidence. It took much longer than anticipated for PROMs workflows to become independent from the *Facilitator*. Audit and feedback of the implementation included routine reporting the proportions of PROMs completed by patients and PROMs acknowledged by staff at team meetings of staff [35].

### Clinician and staff perspectives of barriers and enablers

Ninety surveys were distributed to medical, allied health and nursing professionals currently working in the outpatient clinic who attended staff meetings or scheduled in-service education, during a 4-week period, pre-implementation and following implementation, with 83 and 72 responses received, respectively, at these time points. The demographic characteristics of participants are presented in Table 4.

**Table 4:** Demographic characteristics of staff survey participants

Staff participant	Pre-implementation *N* = 83	Post-implementation *N* = 72
Demographics		
Gender:		
Male	19 (23%)	14 (19%)
Female	64 (77%)	58 (81%)
Age		
20–40 years	24 (29%)	30 (42%)
40–60 years	47 (57%)	42 (58%)
< 60 years	12 (14%)	0 (0%)
Clinician group		
Nursing	53 (64%)	42 (58%)
Medical	19 (23%)	20 (28%)
Allied health	11 (13%)	10 (14%)

Post-implementation, perceived knowledge and understanding of PROMs in clinical practice increased. For example, pre-intervention 82% of doctors, 85% of allied health professionals and 0% of nurses reported good/very good understanding of PROMs, and 41% of doctors, 77% of allied health professionals and 2% of nurses reported good/very good PROM interpretation skills. Post-intervention, 95% of doctors, 70% of allied health professionals and 48% of nurses reported good or very good understanding of PROMs, and 85% of doctors, 50% of allied health professionals and 48% of nurses reported good/very good interpretation skills. Although more staff agreed that the PROM reports were useful and intended to use them in practice following implementation, lack of time remained a barrier to routine use from the nursing and medical perspective. Table 5 shows the pre- and post-implementation responses for each survey item.

**Table 5:** Percentage of staff participants endorsing survey questions before and after implementation

Survey question	Pre (*n* = 83), *n* (%)	Post (*n* = 72), *n* (%)
My understanding of the concept of PROs in clinical practice is very good to fair (vs poor to very poor)	25 (30)	46 (64)
In terms of interpreting PROs, I feel my interpretations skills are very good to fair (vs poor to very poor)	12 (14)	42 (58)
I generally perceive PROs as being very useful to somewhat useful (vs a little useful to I don’t know enough to have an opinion)	38 (46)	52 (72)
I feel my that my colleagues generally perceive PROs as very useful to somewhat useful (vs a little useful to I don’t know enough to have an opinion)	35 (42)	33 (46)
A lack of time would be a barrier to discussing PRO outcomes with my patients in clinic all of the time to most of the time (vs sometimes to none of the time)	58 (70)	43 (60)

## Discussion

This study evaluated implementing PROMs into the routine care of an oncology outpatients’ department using the i-PARIHS framework. The i-PARIHS domains of *Facilitation, Innovation, Recipients* and *Context* were useful for identifying barriers and enablers so that tailored strategies could be developed and put in place. The use of an implementation science framework in a research study offered new insights into the characteristics of implementation in differing *Contexts* and *Recipients* [16]. Using i-PARIHS provided a systematic way to organise observations, provide new insights into how research findings can be translated and how they impact delivery of care in routine oncology health services [16, 38, 39]. This can inform future larger studies, which in turn can be used to improve outcomes in large populations of patients [40].

By using i-PARIHS, we were able to identify, measure and report on how *Facilitation* ameliorated barriers to implementation. Core *Facilitation* strategies included education, awareness raising, and audit and feedback [41–43]. *Facilitation* took a dynamic and flexible approach balanced with the formal structure of *audit and feedback* that acted to inform and regularly engage with *Recipients* (care teams). Similarly, in previous work, Harvey et al. [40] in the FIRE study found that regular meetings were important for implementation to offer goal-focussed practical support, technical assistance and interactive problem solving.

*Facilitation* is a recognised implementation strategy [43], which uses a relational approach to problem solving and may involve trained personnel external to the healthcare context [40]. In our study, the *novice Facilitator* was part of the local staff, but was supported by an *experienced Facilitator* and team to identify and reflect on appropriate strategies [21]. The support of an *experienced Facilitator* was valuable whilst the *novice Facilitator* developed increased awareness, skills and knowledge. Besides providing verbal guidance and problem solving, the *novice Facilitator* had to be physically present in the clinic to support the clinicians, problem solve to make the PROM intervention fit the *Context* and maintain implementation momentum. This demand on the *Facilitator* illustrates the tension between a *Facilitator* doing the work rather than enabling implementation [43]. By keeping up momentum through workflows, the *Facilitator* enabled the experience of using a PROM in clinical practice for staff [44, 45]. However, for sustainability, the intervention would need to be fully integrated into routine workflows before scaling up.

The *Facilitator’s* implementation strategies resulted in a shift in staff perceptions about the usefulness of PROMs, their knowledge about PROMs and how they could use them practically to improve their clinical care. Interestingly, whilst nursing knowledge and confidence improved markedly post-implementation, allied health professionals reported somewhat lower knowledge and confidence post-implementation. Whilst the reason for this is unclear, the very high self-reported knowledge and confidence pre-implementation may have reflected lack of experience applying PROMs within this complex patient group, with more conservative estimates after experiencing the PROM implementation. Additionally, the doctors and nurses may have had more exposure to the PROMs which may have increased their knowledge and confidence. Staff engagement with PROMs has been identified as essential for implementation success [29].

Staff surveys showed that the acceptability of PROMs increased from pre- to post-implementation. The construct of acceptability has been described in the literature as dynamic and changing as *Recipients* experience an innovation/intervention and gain a deeper understanding of “what” is involved [4], and this matched our experience in the case study. In the post-implementation survey sample, the perceived “usefulness” (*relative advantage*) of PROMs increased as clinicians started to report that they understood better the value of PROMS and reduced barriers to PROMs use [4, 21, 46].

We also found that the collection of formative data in our case study was essential to enable successful facilitation. Staff responded to quantitative data of completion rates and staff acknowledgement rates with accompanying observational data, such as changes to workflows in response to events taking place in clinical areas. This use of data assisted in giving all *Recipients* a deeper understanding of the factors at play in the *Context*, and insight into the importance of the role staff have in the facilitating implementation.

Implementation science theories, such as i-PARIHS, can provide rich data to support and expand existing frameworks for PROMs. For example, Van der Wees and colleagues described a sequence of steps for choosing and implementing PROMs (goal setting, selecting PROs and PROMs, developing and testing of quality indicator(s), and implementing and evaluating the PROM(s) and indicator(s)) [8]. Our case study shows that it may be worthwhile to combine PROM-specific frameworks with implementation science theories to better characterise the ways in which PROM attributes (e.g. validity and reliability) may interact with the implementation process. Implementation science theories systematise barriers so they can be linked to the broader implementation literature [47], directly link barriers to evidence-based support strategies [48] and provide a way to examine contextual factors influencing implementation (e.g. organisational readiness to change and clinic culture) [48].

Whether implementation is successful or not, there is much to learn when it is systematically described, and this knowledge is disseminated [49].

## Limitations

We recognise some limitations with the evaluation. Data used to inform the analysis of the i-PARIHS constructs included observations collected by the *Facilitator* (NR) in site journals and field notes. This may be biased both by her perspective as a clinical trial nurse within the study context, and as the *Facilitator*, and are intrinsically subjective judgements. There is a balance in an implementation evaluation between the deep knowledge of context available to an internal *Facilitator*, and the risk of bias inherent in holding this tacit knowledge. Mechanisms to increase objectivity included contemporaneous notes, triangulation of data sources, reflexive monitoring with a research team, including members internal and external to the *Context*, and regular reflection with an *expert Facilitator* external to the project team. Staff surveys were anonymous, and used as cross-sectional data, and it is unknown where there is some overlap between respondents at both time points. The exact numbers of staff surveys distributed to each professional group was not able to be accurately captured, only the total number distributed due to the non-identifiable approach to data collection.

The detailed description of the *Recipients* and *Context* highlights the importance of local factors for implementation success, which means that findings may not be generalisable to other clinics or settings. However, these findings could inform approaches to implementation in other settings as the enablers and barriers had consistency with the findings of the initial scoping review [14].

## Conclusions

The i-PARIHS framework was useful to systematically inform and iteratively plan the implementation of PROMs into routine oncology care. The four core i-PARIHS constructs (*Facilitation*, *Recipients*, *Innovation* and *Context*) all needed to be addressed to allow successful implementation, with *Facilitation* having a particularly important role moderating the other domains of *Recipients*, *Innovation* and *Context*. In this study, the PROMs intervention was acceptable to many staff, but complex barriers mean that sustainability may not have been achieved, and teams may struggle to maintain PROMs once the *facilitation* role has been withdrawn. Further research into efficient implementation and sustainability of PROMs is required to ensure lasting benefits.
